# Probing Bacterial Interactions with the *Schistosoma mansoni*-Killing Toxin Biomphalysin via Atomic Force Microscopy and Single Molecule Force Spectroscopy

**DOI:** 10.3390/toxins17060269

**Published:** 2025-05-27

**Authors:** Jihen Zouaoui, Pierre Poteaux, Audrey Beaussart, Nicolas Lesniewska, David Duval, Jérôme F. L. Duval

**Affiliations:** 1 Université de Lorraine, CNRS, LIEC, F-54000 Nancy, France; jihen.zouaoui@univ-lorraine.fr (J.Z.); audrey.beaussart@u-bordeaux.fr (A.B.); nicolas.lesniewska@polito.it (N.L.); 2 IHPE, CNRS, IFREMER, Université de Montpellier, Université Perpignan Via Domitia, F-66860 Perpignan, France; pierre.poteaux@univ-perp.fr

**Keywords:** biomphalysin, AFM, SMFS, interaction, *M. luteus*, *E. coli*, *Biomphalaria glabrata*, adhesion

## Abstract

Recent work has identified biomphalysin (BM) protein from the snail *Biomphalaria glabrata* as a cytolytic toxin against the *Schistosoma mansoni* parasite. Ex vivo interactome studies further evidenced BM’s ability to bind bacterial outer membrane proteins, but its specific antibacterial mechanisms and selectivity remain unclear. Accordingly, this study aims to elucidate the interaction between BM and two model bacteria with distinct cell surface architectures: *Escherichia coli* (Gram−) and *Micrococcus luteus* (Gram+). Employing a multiscale approach, we used in vivo single-molecule force spectroscopy (SMFS) to probe molecular interactions at the single cell level. Combined with cell aggregation assays, immunoblotting and Atomic Force Microscopy (AFM) imaging, SMFS results evidenced a selective interaction of BM from snail plasma with *M. luteus* but not *E. coli*. Exposure of *M. luteus* to BM compromised cell surface integrity and induced cell aggregation. These effects correlated with a patch-like distribution of BM on *M. luteus* reminiscent of pore-forming toxins, as revealed by the anti-BM antibody-functionalized AFM tip. Overall, this work highlights the utility of SMFS in dissecting host–pathogen molecular dialogs. It reveals BM’s selective action against *M. luteus*, potentially via surface clustering, and it shows spatially heterogeneous responses to the toxin within and between individual cells.

## 1. Introduction

*Biomphalaria glabrata*, a tropical planorbid snail, is the major intermediate host for the *Schistosoma mansoni* parasite, one of the most important human-infective schistosome species. In 2022, more than 250 million people worldwide needed a treatment for schistosomiasis (also called bilharzia), the disease caused by a schistosome parasitosis [1]. *B. glabrata* has been the subject of numerous studies due to its role in the transmission of the bilharzia-responsible agent. These studies aimed at understanding not only how the immune system of *B. glabrata* responds to *S. mansoni* invader [2,3,4] but also how it coordinates the defense against the infection caused by other trematode species [5,6,7] or non-specifically acting prokaryotic organisms [8]. Interestingly, *B. glabrata* is also sensitive to some bacterial infections [9]. In the context of *B. glabrata*’s immune response analysis, the hemolymph has been the focus of much attention. This fluid consists of distinct cellular and humoral compartments, the former containing hemocytes and the latter defining the plasma which is mainly composed of free hemoglobin (counting for 80% of the protein mass [10,11]) and humoral factors involved in the immune function [3,12]. The panel of immune factors includes various lectin-domain proteins, such as Galectin-Related Proteins, C-type lectin-Related Proteins, Fibrinogen-Related Proteins [13,14], thioester-containing proteins (TEP) [15,16,17], and biomphalysins (hereafter generically denoted as BM) which form a family of 23 genes of β-Pore-Forming Toxins (β-PFTs) [18,19]. Like β-PFTs, biomphalysins feature a specific structural prediction and a capacity to bind to and induce lysis of *S. mansoni* sporocysts [18]. This protein family forms a group of humoral factors that play a lytic effector role, unlike other plasma lectins which primarily operate in agglutination and opsonization processes [20,21,22]. Among the β-PFTs, biomphalysins are related to the family of aerolysin, a bacterial toxin produced by *Aeromonas hydrophila* that targets glycosylated and glycosylphosphatidylinositol (GPI)-anchored membrane proteins of eukaryote cells [23,24,25]. Accordingly, this toxin forms stable transmembrane β-barrels, which results in the permeation of the membrane of targeted cells and their subsequent lysis. Through several horizontal gene transfer events, a polyphyletic dispersion of aerolysin-like genes led to the acquisition of this protein type by a large diversity of eukaryote organisms, e.g., Chlorobionta, and, among Metazoa, Cnidaria and both Protostomian and Deuterostomian Bilateria [26,27,28]. Such a phylogenetic spread allowed for a great diversification of protein sequence [29] and the contribution of aerolysin-like proteins to new biological functions in connection with the recognition of various molecular targets [30,31].

On the basis of an interactome study, Tetreau et al. [32] demonstrated the recognition of outer membrane proteins of different cell types by various biomphalysins, e.g., *S. mansoni* and *Echinostoma caproni* miracidia, *Saccharomyces cerevisiae* yeast, and *Escherichia coli* and *Micrococcus luteus* bacteria. This ex vivo approach allowed the identification of biomphalysins as potential pathogen-interacting proteins. To the best of our knowledge, biomphalysins have been mainly investigated so far through analyses of their evolution history and expression [19], and studies on some of their functions [18,32]. However, the way the biomphalysin immune factor specific to *B. glabrata* interacts with the surface of cells it can recognize remains poorly documented from a physicochemical point of view. In particular, the properties of adhesion of *B. glabrata* biomphalysins on the surface of recognized cells have not yet been measured at the relevant single-cell or molecular levels. These properties include the magnitude and spatial range of the interaction forces operating between biomphalysins and cell surface receptors, and the way biomphalysins’ adhesion features distribute all over the surface of the cells they target. More generally, understanding at the molecular level the forces that drive host–pathogen interactions is a prerequisite for combating pathogenesis and infection, and identifying new therapeutic targets [33,34,35,36].

In this context, single-molecule force spectroscopy (SMFS)—a technique derived from Atomic Force Microscopy (AFM)—has emerged as a valuable and powerful tool to examine biomolecular interactions with unprecedented spatial resolution [37,38,39,40,41]. AFM stands out as one of the few current techniques capable of detecting, locating and manipulating individual molecules on living cells, providing nanoscale-resolution topographic images of cell surface features while preserving the integrity of the sample surface [37,42,43]. Unlike other microscopy techniques, AFM imaging of cell surfaces is achieved without staining, marking or drying, under physiological liquid conditions, and it uses a cantilever supporting a sharp nanometric tip that is moved in all three spatial directions to scan a cell surface of interest [36,37,38,40,41,42,43]. The asperities/depressions of the cell surface are tracked via the deflection of the cantilever caused by the forces felt by the tip when approaching/leaving the surface, and this deflection is monitored via the reflection of a laser beam toward a position-sensitive photodiode [37]. Based on the same measuring principle, the SMFS technique makes use of functionalized AFM tips to map the distribution of specific molecules located on living cells [40,44]. To that end, the tips are coated with dedicated ligands and scanned over the cell surface to measure and map the surface receptor–ligand forces upon retraction of the tip from the cell surface [41]. Such molecular force measurements have provided new insights into the organization of cell surface adhesins and their role in cell binding to various biotic and abiotic supports [45,46,47,48,49,50]. For the sake of illustration, SMFS studies revealed a force-induced formation and expansion of nanodomains of Als5p adhesins on the surface of *Candida albicans*, a mechanism that possibly triggers cell adhesion [51]. SMFS experiments also evidenced that staphylococcal adhesins like SdrG, ClfA, and ClfB can bind to their protein ligands according to multistep dock, lock, and latch (DLL) mechanism, leading to interaction forces (~2 nN) comparable to those of covalent bonds [52,53]. SMFS also made it possible to map the distribution of outer membrane cytochromes in *Shewanella oneidensis* [54], to address the dynamics of interaction between self-associating auto-transporter (SAAT) adhesins of bacteria [45], or to decipher the nanomechanical properties of various bacterial surface appendages, including pili or fimbriae involved in *E. coli* surface colonization and biofilm formation [47].

Given the aforementioned elements, the objective of this work is to investigate, using AFM and SMFS, the effects and adhesion properties of plasmatic biomphalysins from *B. glabrata* on Gram-negative *E. coli* and Gram-positive *M. luteus* bacteria. This investigation will be conducted at the molecular scale from the single-cell to multi-individual levels. The choice of these bacteria as cellular candidates targeted by biomphalysin is motivated by the proteomic profiling analysis previously reported by our group [32]. AFM imaging is performed here to address the modifications of the surface features of the selected bacteria after their incubation with biomphalysin, and to evaluate the corresponding changes in cell surface roughness over time. The SMFS measurements detailed in this work are performed by means of AFM tips functionalized with the anti-biomphalysin antibody. AFM and SMFS analyses are further complemented by a cell aggregation assay and immunoblotting measurements. Overall, despite the *non-specific* interactions occurring between functionalized AFM tip and cell surfaces decorated or not by biomphalysin, AFM and SMFS data indicate a positive correlation between the following: (i) biomphalysin-mediated *M. luteus* aggregation, (ii) *M. luteus* surface damage and an increase in cell surface roughness, and (iii) molecular interactions of *M. luteus* surface components with biomphalysin distributed on the *M. luteus* surface according to patch-like patterns. The current work fills a gap in the literature in the sense that it provides an in vivo AFM- and SMFS-based assessment, with a molecular spatial resolution, of the damage caused by biomphalysin on cell surface targets at the single-cell level. It further reports a rationale for the binding force of biomphalysin to Gram-positive and Gram-negative bacteria and its distribution across their surface.

## 2. Results and Discussion

Before presenting and discussing the Atomic Force Microscopy (AFM) imaging data and Single Molecule Force Spectroscopy (SMFS) results (Section 2.2 and Section 2.3, respectively), we first provide in Section 2.1 macroscopic cell aggregation data which aim to support (or refute) the existence of interaction between *E. coli*/*M. luteus* and plasmatic biomphalysin from *B. glabrata*. Among the 23 biomphalysin genes identified in *B. glabrata*, those of biomphalysins 1 and 2 (hereafter termed as B1/2) exhibited the highest transcription levels [19]. Furthermore, recent findings have identified B1 and B2 as the most abundant biomphalysin proteins in *B. glabrata* plasma, and they are particularly abundant in plasma as compared to other snail tissues [55].

### 2.1. Bacterial Aggregation Mediated by B. glabrata Plasma Components, Including Biomphalysin

*B. glabrata* plasmatic compartment contains many different immune molecules, including lectins [4,13] and fibrinogen related proteins (FREPs) [13,22,56,57], thioester containing proteins (TEPs) [12,16,17], and biomphalysins [18,19], the latter being the main humoral protein effector known to have a lytic activity in the planorbid snail *B. glabrata* [3,18]. Some humoral factors like FREPs and other lectins are further able to bind Gram-negative and Gram-positive bacteria [32,56,58].

**Figure 1:**
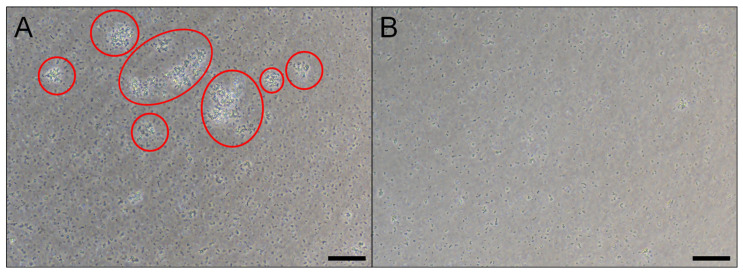
Aggregation potential of *B. glabrata* plasma components, including biomphalysin, against *M. luteus*. Optical images showing the aggregation of *M. luteus* after exposure to ultracentrifuged *B. glabrata* plasma (**A**), as compared to the control situation of cell incubation in CBSS (Chernin’s balanced saline solution) (**B**). Scale bars: 50 µm. Red circles point out the presence of cell aggregates. Plasma and cell culture were prepared and cell aggregation assays performed along the lines detailed in Materials and Methods (Section 3.1, Section 3.2, Section 3.3 and Section 3.4).

Figure 1 evidences that ultracentrifuged plasma from *B. glabrata*, which contains biomphalysins, has an aggregating potential against *M. luteus* bacteria, and this potential is correlated with the ability of some immune factors like lectins and biomphalysins to bind *M. luteus* cells [14,19,32]. This result is consistent with the reported binding of some potential biomphalysin partners such as thioester containing proteins (TEP) to *M. luteus* [17]. Additional experiments showed the absence of aggregating potential of ultracentrifuged plasma against *E. coli* under the same exposure conditions than those adopted for *M. luteus*, which contrasts with the existence of interaction between *E. coli* and proteins from ultracentrifuged plasma of *B. glabrata* evidenced in ref. [32]. This finding suggests that (i) *E. coli* and *M. luteus* may interact with the same protein partner(s) *albeit* at different magnitudes/strengths, or that (ii) *M. luteus* and *E. coli* interact with distinct proteins according to different mechanisms, one leading to cell aggregation and the other not. Hypotheses (i) and (ii) are not mutually exclusive, and they indeed align with previous findings: an interactome analysis, conducted with both bacterial strains exposed to plasma, revealed distinct proteins interacting with *M. luteus* and *E. coli*, encompassing lectins and biomphalysins [32]. In particular, an extensive array of peptide sequences, matching biomphalysins B1 (5 sequences), B2 (4 seq.), B3 (5 seq.), B4 (3 seq.), B5 (2 seq.), B6 (2 seq.) and B7 (3 seq.), were detected on *M. luteus* following plasma exposure [19,32].

**Figure 2:**
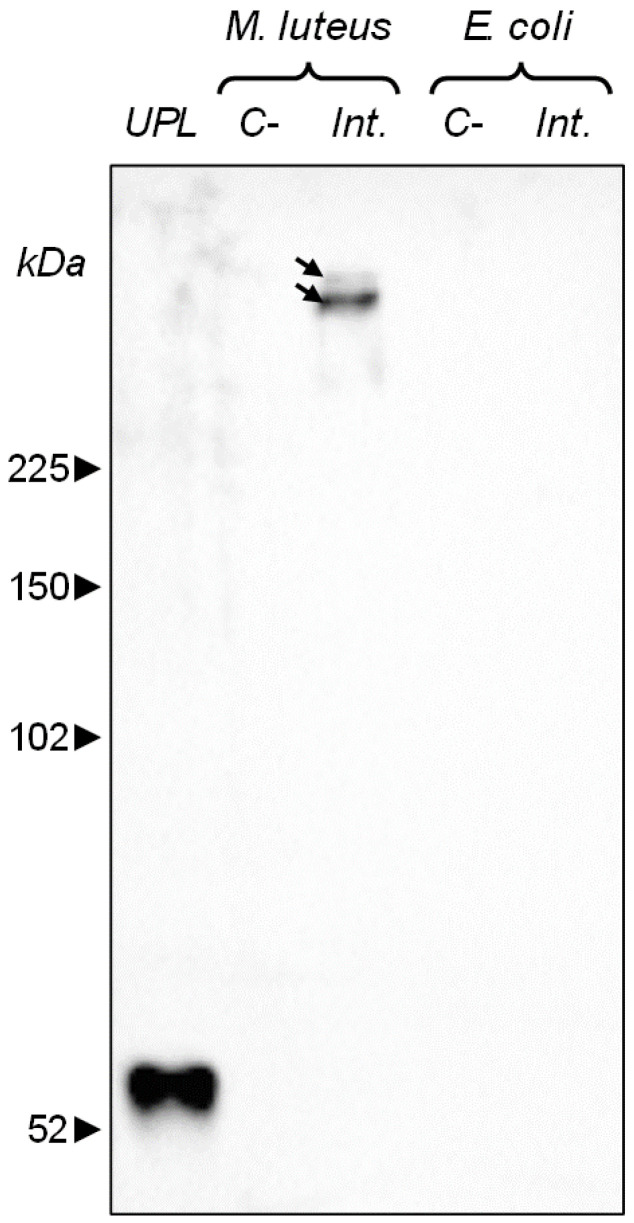
Immunoblotting analysis of biomphalysins 1/2 interactions with *M. luteus* and *E. coli*. The interaction between biomphalysins 1/2 and *M. luteus* (Gram-positive) was monitored after incubation of the cells with ultracentrifuged plasma of *B. glabrata*. Ultracentrifuged plasma (UPL) was also put in contact with *E. coli* cells (Gram-negative), and immunoblotting analysis did not reveal binding for these cells. C-: negative control, cells were incubated with CBSS for 2 h instead of ultracentrifuged plasma. Int.: interaction condition, cells (either *M. luteus* or *E. coli*) were incubated with ultracentrifuged plasma prior to immunoblotting. Two bands of high molecular weight are detected in the plasma-interacting condition (arrows), suggesting that the protein is present in an oligomeric form. Immunoblotting was performed as detailed in Materials and Methods (Section 3.1, Section 3.2, Section 3.3 and Section 3.4). UPL: ultracentrifuged plasma of *B. glabrata*.

Biomphalysin 1 is the most transcribed biomphalysin gene in a whole naive non-infective snail, and it is an abundant protein in snail plasma [55]. Moreover, with biomphalysin B2, it is one of the biomphalysins detected in plasma with the best sequence coverage obtained after proteomic analyses [55]. The two proteins B1 and B2 have very similar peptide sequences and, in proportion to the total number of peptides that can be detected for each of the two sequences, quite few peptides are specifically unique to one or the other sequence [55]. Accordingly, given that both Western blotting (this section) and SMFS experiments (Section 2.3) make use of the same anti-biomphalysin B1 antibody (cf. details in Section 3.3), it is virtually impossible to differentiate B1 and B2 proteins. Native forms of biomphalysin 1/2 is detectable at a size of approximately 55 kDa in ultracentrifuged plasma (Figure 2) [55]. A 2 h exposure of *E. coli* to *B. glabrata* plasma does not result in a detectable binding of B1/2, as determined by Western blotting (Figure 2). On the opposite, *M. luteus* exposure to ultracentrifuged plasma of *B. glabrata* induces a significant change in the size of B1/2, as evidenced by the band detected above the 225 kDa size marker (cf. arrows in Figure 2). Scrutinizing the literature on proteins that are representatives of β-pore forming toxins (β-PFT), the above increase in the size of biomphalysins can be attributed to a denaturation-resistant oligomerization: indeed, β-barrel pores, once formed, are resistant to the denaturation sets for a SDS-PAGE [55,59,60,61,62,63,64,65,66] and they can thus be identified through Western blotting. The high molecular weight bands could also originate from mixed homo- or hetero-oligomeric complexes of different biomphalysin variants. Indeed, biomphalysin is part of a multigenic family in *B. glabrata* and multiple isoforms are expressed constitutively.

The detection of biomphalysin B1 (and B2) binding on *M. luteus* and, more importantly, the evidence of a high molecular size of bound biomphalysins, strongly indicate a biological activity of biomphalysins 1/2 against this bacterium species, which agrees with conclusions of ref. [32]. As biomphalysins are the only plasmatic factors in *B. glabrata* known to have by themselves a lysis potential [13,19,67], we expect that the plasma has the potential to either induce a lysis of *M. luteus* after binding of biomphalysins or, at least, mediate a modification of the native biophysicochemical properties of the bacterial surface. In the developments below, we report AFM and SMFS measurements to obtain further insights into the biomphalysins-*M. luteus* interactions revealed in Figure 1 and Figure 2 by cell aggregation assay and immunoblotting. Experiments are also performed on *E. coli* to address whether or not the absence of interaction with biomphalysins 1/2 evidenced at the macroscopic scale (Figure 2), is confirmed by AFM and SMFS measurements with molecular scale resolution at the (multi-)individual cell level.

### 2.2. Cell Surface Roughness Measurements by AFM Imaging

**Figure 3:**
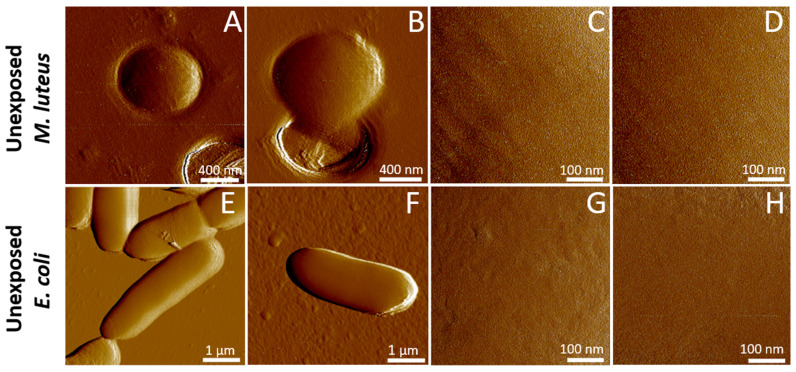
Representative AFM images of unexposed *M. luteus* and *E. coli* bacterial cells measured in aqueous medium. Low (**A**,**B**,**E**,**F**) and high (**C**,**D**,**G**,**H**) magnification peak force error images of unexposed *M. luteus* (**A**–**D**) and *E. coli* (**E**–**H**) bacteria in buffer solution.

Figure 3A–D show a set of illustrative AFM images obtained in Peak-Force Tapping Mode on *M. luteus* in phosphate-buffered saline (PBS 1X) solution, prior to their exposure to ultracentrifuged plasma from *B. glabrata*.

Figure 3A–D feature the presence of *M. luteus* spherical bacteria (0.9 to 1.1 µm in diameter) trapped in a 1.2 µm microporous membrane used for cell immobilization, as required for AFM imaging (cf. details in Section 3, Section 3.5 and Section 3.6). The surface of *M. luteus* cells appears smooth, free of significant asperities and depressions, with a Root Mean Square (RMS) roughness of 2.1 ± 0.8 nm (value derived from the analysis of *N* = 10 cells), in agreement with reported RMS roughness data for bacterial surfaces [68,69,70]. In addition, the AFM images evidence that cell surface morphology does not significantly differ among analyzed cells, thereby suggesting the absence of distinct cell surface phenotypes that could subsequently mediate different responses to- and interactions with- biomphalysin. Figure 3E–H show the counterpart of Figure 3A–D for native *E. coli* cells before exposure to biomphalysin, and the *E. coli* cells are immobilized here on a glass side pre-coated by (cationic) polyethyleneimine (PEI, cf. details in Section 3.5 and Section 3.6). Values of cell surface RMS roughness 3.0 ± 0.9 nm (*N* = 12) are also in line with literature data [68,69,70]. Imaged *E. coli* cells display an expected rod-like shape, 3 to 4 µm in length, and similarly to *M. luteus*, AFM does not reveal remarkable surface structures nor protruding cell surface appendages, e.g., apparent fimbriae, exopolysaccharides or pili.

**Figure 4:**
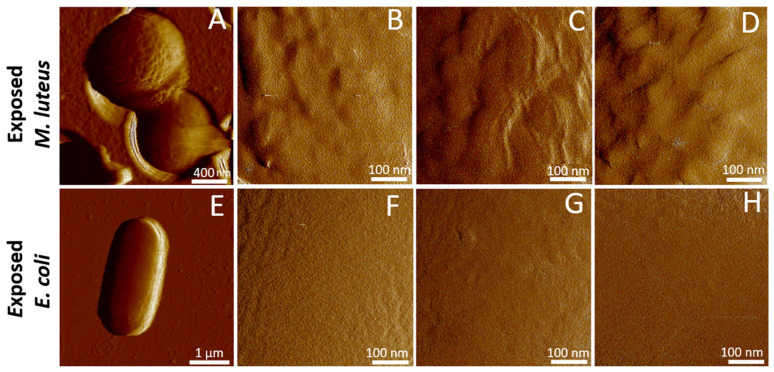
Representative AFM images of *M. luteus* and *E. coli* bacterial cells measured in aqueous medium, after cell exposure to ultracentrifuged plasma of *B. glabrata*. Low (**A**,**E**) and high (**B**–**D**,**F**–**H**) magnification peak force error images of *M. luteus* (**A**–**D**) and *E. coli* (**E**–**H**) cells after 5 min exposure to ultracentrifuged plasma containing biomphalysins (cells were imaged in buffer solution after rinsing).

**Figure 5:**
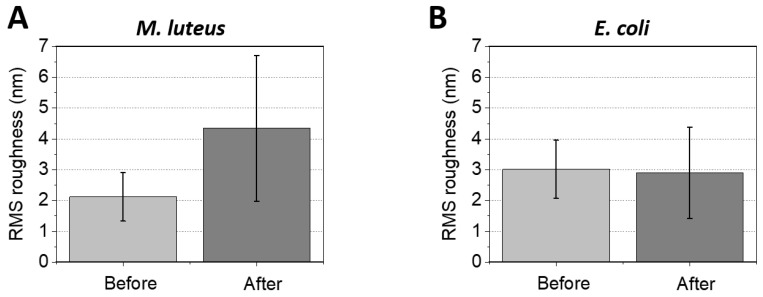
Effect of the exposure to plasma containing biomphalysins on the surface roughness of *M. luteus* and *E. coli*. (**A**,**B**) Histograms showing changes in RMS surface roughness evaluated from AFM height images of *M. luteus* (**A**) and *E. coli* (**B**) before (light gray) and after (dark gray) exposure to *B. glabrata* plasma. Data for *M. luteus* (**A**) originate from measurements on *N* = 10 unexposed cells and *N* = 14 exposed cells, and for *E. coli* on *N* = 12 unexposed cells and *N* = 9 exposed cells. Statistical analysis using a Student’s *t*-test yielded a *p*-value of 0.0022 for *M. luteus*, confirming a significant increase in its surface roughness as a result of exposure to plasmatic biomphalysin. In contrast, performing a similar Student’s *t*-test for *E. coli*, we obtained a *p*-value of 0.84 (>0.05), which supports that the difference in cell surface roughness before and after exposure to biomphalysin is statistically not meaningful for *E. coli*.

Figure 4A–D collect representative AFM images of *M. luteus* after a 5 min exposure to 50 μL of biomphalysin-containing ultracentrifuged plasma from *B. glabrata*, followed by a washing with PBS to remove toxins which only weakly interacted with cell surface or were in excess in solution (cf. Section 3.5 and Section 3.6). In agreement with the immunoblotting analysis of Figure 2, qualitative inspection of Figure 3A–D (before treatment) and Figure 4A–D (after treatment) shows that the surface of *M. luteus* did interact with the plasma, and that this interaction lead to the apparition of local inflammation spots taking the form of multiple, elongated and possibly interconnected blisters or granules with size as large as 100 nm. These blisters or granules further seem to distribute homogeneously over the cell surface. While many of the analyzed cells have experienced the above surface damage as a result of the action of the plasma containing biomphalysins, others remain unaffected. In other words, there is a certain heterogeneity in the response of *M. luteus* to plasma exposure. The latter feature was also observed for other bacterial species in response to various molecular and particulate stressors, like metallic (nano)colloids [70] or antimicrobial peptides [71,72]. However, the action modes of these stressors seem to differ from the one of plasma and, by extension, biomphalysins, as we did not observe here the formation of open holes or craters at the surface of the cells, nor obvious signs of cell swelling or hypertrophy. Overall, the 5 min exposure to 50 μL ultracentrifuged plasma resulted in an increase in the RMS surface roughness of *M. luteus* from 2.1 ± 0.8 nm to 4.3 ± 2.4 nm, as illustrated in Figure 5.

The heterogeneous response of *M. luteus* cells to plasma exposure, as captured by AFM imaging, can be attributed to a combination of possible factors related to the inherent variability within the bacterial population. Specifically, bacterial populations are generally not homogeneous and individual cells can exist in different physiological states (e.g., different stages of the cell cycle, metabolic activity levels). In the current context, such variations can influence how susceptible a cell is to plasma-induced damage and subsequent changes in its surface properties. Even within a clonal population, there can be subtle differences in the cell wall composition and organization of the outer layers. In turn, these variations can lead to differential interactions with the plasma and the AFM tip. We stress that *M. luteus* were precultured overnight and then cultured until a DO of ca. 0.3 was achieved, prior to exposure to plasma. Harvesting bacteria in their exponential growth phase ensures the most relevant physiological state of the cells for probing BM interactions. However, as the cultures were not synchronized, individual bacteria may not be in the same cell cycle, which possibly influenced their susceptibility to the toxin-containing plasma.

It is well-established that cell responses to stressors can be transient and reversible, provided that the stressor is eliminated or that compensatory cellular mechanisms are activated over time in the presence of the stressor. In certain cases, damaged cells may even fully recover their functional capabilities while in others residual cell damage may persist [73]. To address how the surface damage described in Figure 4A–D and caused on *M. luteus* by the biomphalysins-containing plasma fits into that picture, we measured over time AFM images of individual cells in contact with 4 mL PBS solution and 10 μL of ultracentrifuged plasma (Figure S1 in Supplementary Materials, SM). Results highlight (i) a rapid increase in the RMS cell surface roughness from ca. 3.4 nm to 6.1 nm over the first 30 min of the experiment, followed by a constant plateau value observed between ca. 1 and 2 h after the start of the experiment, and (ii) a persistence of the blisters/granules invoked in Figure 4A–D and formed at the so-rendered raspberry-like surface of *M. luteus*. Accordingly, the action mode of biomphalysin can be qualified as fast (timescale of few minutes), and the bacteria do not have the possibility to repair over time the plasma-mediated damage of their surface under the conditions of Figure S1. AFM imaging of cell surface domains down to few nm^2^ dimension did not reveal the presence of nm-sized (pre-)pore surface structures that are associated with the action of β-Pore Forming Toxins (PFT) on cell membranes [74]. The absence of classical pore-like structures revealed by AFM may reflect a different mechanism than canonical pore formation, and differences in bacterial surface structure (Gram-positive *vs.* Gram-negative) likely also contribute.

This finding, however, does not necessarily mean that such (pre-)pores are absent from the cell surface because the convolution of the cell surface topography by the AFM tip geometry (20–30 nm radius at the apex, cf. Section 3.5 and Section 3.6) can make the technique blind to such nm-sized surface protein organization and to oligomeric forms of the pore-forming toxin at the cell surface. In addition, the molecular mechanisms by which β-PFT act on Gram-positive bacteria that feature a (rigid) peptidoglycan surface layer remain unclear, especially with regard to the very location of the toxin oligomerization process (i.e., at the cell surface or deeper in the cell envelope, once toxin monomers have crossed the peptidoglycan layer) [75]. Last, we report in Figure 4E–H AFM images of *E. coli* after 5 min incubation with 50 μL biomphalysin-containing ultracentrifuged plasma (to be compared with the images in Figure 3E–H prior to exposure to the toxin). Unlike for *M. luteus* exposed under similar conditions, we find that RMS roughness of *E. coli* surface (2.9 ± 1.8 nm, *N* = 9, Figure 5) and associated cell surface morphology are largely insensitive to treatment by ultracentrifuged plasma. This result, which contrasts with those detailed above for *M. luteus*, is consistent with cell aggregation assays and immunoblotting data (Figure 1 and Figure 2).

The observed *M. luteus* aggregation may arise from the action of the biomphalysins directly, or from other plasmatic immune factors. The correlation between the aggregation process (Figure 1), biomphalysin binding and oligomerization (Figure 2) suggests complete plasma activity toward *M. luteus* and not *E. coli* bacteria, despite the capacity of certain plasmatic factors to bind to the latter bacterial species [17,32]. Recent findings [3] support the existence of both direct and indirect mechanisms of parasite recognition in *B. glabrata*, where proteins like BgFREPs (fibrinogen-related proteins involved in pathogen recognition) and BgTEP1 (a thioester-containing protein implicated in opsonization and immune complex formation) interact together and with biomphalysin to mediate immune responses. Further looking at the work by Gorbushin [58], we may thus keep in mind the hypothesis of a complement-like pathway involving lectins and pore-forming toxins. Following this hypothesis, the aggregation of *M. luteus* could be due to binding proteins such as lectins, and BM binding would only constitute the terminal effector interaction. Even though the interaction between plasmatic proteins involved in *B. glabrata* immunity is not known yet, some BM-partner candidates related to complement system proteins seem to interact together (BgFREP/BgTEP) [3].

Given that biomphalysins are considered as the sole lytic effectors within the plasma, and further considering the demonstrated ability of B1/2 to bind to *M. luteus* but not *E. coli* (cf. Figure 2 and prior interactome study in [32]), we can establish a causal relationship between B1/2 binding and bacterial surface alteration (Figure 4 and Figure 5). In the following developments, we have recourse to AFM operated in Single Molecule Force Spectroscopy (SMFS) to evaluate the distribution of biomphalysin over the surface of *M. luteus* and *E. coli* at the molecular scale.

### 2.3. SMFS Interaction Force Measurements

**Figure 6:**
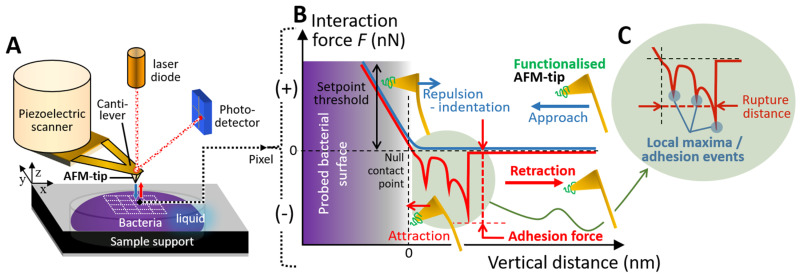
Schematic representation of single molecule force spectroscopy (SMFS) measurements between an AFM tip functionalized with anti-biomphalysin antibody and a bacterium immobilized on a substrate (sample support). (**A**) Details of sample environment and scheme of AFM measurement principle. The vertical and horizontal movements of the AFM tip as it scans the cell surface are monitored via the reflection of a laser beam from the cantilever to a position-sensitive photodetector that registers the vertical and lateral displacements of the laser. (**B**) Illustration of typical force *versus* separation distance profile measured on the approach (blue curve) and retraction (red curve, of specific interest in this work) of the AFM tip to- and from- the surface, respectively, and definition of the nomenclature adopted in this work. In the example, the approach force curve corresponds to a repulsion (i.e., the measured force is positive) between the AFM tip and the cell surface prior to- and after- the cell-to-tip contact. After the cell-to-tip contact, the AFM tip indents (penetrates) the cell surface. (**C**) Zoom of local force maxima detected in the retraction force *vs.* distance curve featuring successive adhesion events upon progressive withdrawal of the AFM tip from the cell surface. See text for details.

SMFS experiments were conducted in Force Volume (FV) mode to investigate the interactions between bacterial cells (*M. luteus* and *E. coli*) and biomphalysins B1/2. To that end, AFM tips were functionalized with anti-biomphalysin 1/2 antibodies, enabling specific recognition of the targeted BM proteins at the probed cell surfaces. Prior to SMFS measurements, bacteria were immobilized on a solid substrate (*E. coli*) or in a porous membrane (*M. luteus*), and subsequently exposed for 5 min to biomphalysin (B1/2)-containing ultracentrifuged plasma from *B. glabrata* along the lines set forth in Section 3.5 and Section 3.6. Force measurements were then performed by approaching and retracting the functionalized tip from the cell surface, resulting in the acquisition of force curves at each point (pixel) within a defined area of the cell surface. Each cell was scanned over a 500 nm × 500 nm area, generating a 32 × 32 pixels map. At each pixel, a pair of force *versus* distance curves was recorded, one during the approach of the AFM tip towards the cell surface, and another during the retraction of the tip (Figure 6). All experiments were carried out under controlled liquid conditions at room temperature, and the reader is referred to Section 3.6 and Section 3.7 for additional details on SMFS experiments and AFM tip functionalization procedure, respectively. As schemed in Figure 6, measured retraction force-distance curves often exhibit a sawtooth pattern, potentially resulting from the sequential unfolding of cell surface proteins that specifically bind the functionalized AFM tip or from multiple attachments/detachments of cell surface components to/from the AFM tip [76,77,78,79]. The processing of all SMFS force curves was performed according to the methodology outlined in Section 3.8. Briefly, the analysis consisted in identifying and counting the number of tip-to-surface **adhesion events** in SMFS force curves upon detection of local force maxima (Figure 6). Depending on analyzed systems, the number of local force maxima may also inform on the number of sequential events of biomolecule unfolding that occur when retracting the functionalized tip from the cell surface. Furthermore, the force associated with the last detected adhesion event, termed as **adhesion force**, was specifically evaluated together with the tip-to-surface separation distance at which the tip was detached from the cell surface, hereafter called **rupture distance** (Figure 6). In turn, the above analysis of SMFS measurements lead to the generation of (32 × 32 pixels) maps featuring the spatial distributions of the number of adhesion (or biomolecule unfolding) events, the adhesion force and the last rupture distance over the scanned surface area of selected cells. The mean frequency distributions of these parameters determined for different cells (typically between *N* = 5 and *N* = 18, depending on examined conditions) could be evaluated in the form of histograms.

**Figure 7:**
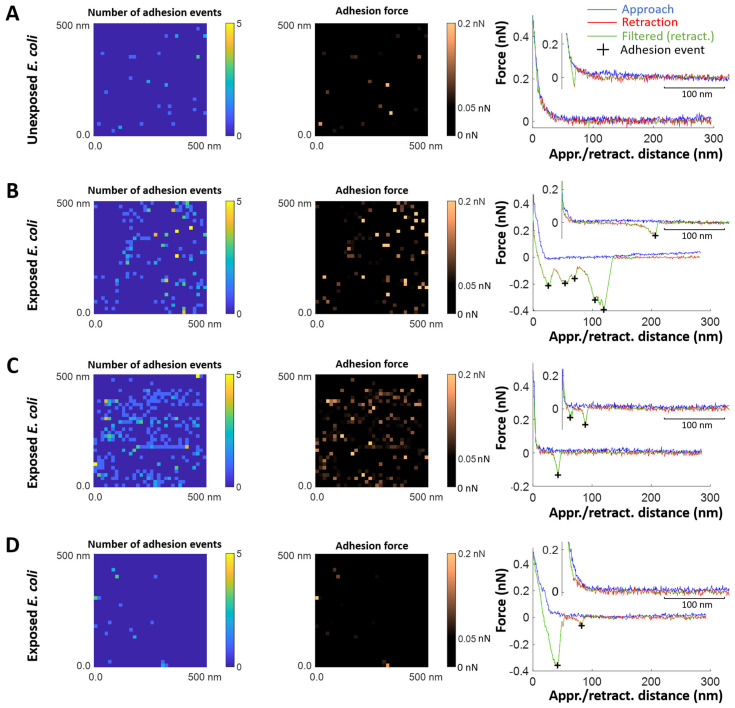
Adhesion properties of anti-biomphalysin (B1/2) antibody on *E. coli* before and after exposure to ultracentrifuged plasma from *B. glabrata*. Representative SMFS measurements obtained in Force Volume mode with anti-biomphalysin (B1/2) antibody-functionalized AFM tip for different *E. coli* cells unexposed (**A**) and exposed (**B**–**D**) to biomphalysins-containing ultracentrifuged plasma from *B. glabrata.* Columns 1 and 2: maps of the number of adhesion events and adhesion force, respectively. Column 3: representative force curves measured in the approach (blue) and retraction (red) stage of the AFM tip. The green curves correspond to a mean-shift smoothing of the raw retraction force curves as performed in the data postprocessing step for identification and evaluation of the relevant tip-to-cell adhesion features. See text for details.

We show in Figure 7 illustrative SMFS results obtained for different *E. coli* cells unexposed (Figure 7A) and exposed (Figure 7B–D) to biomphalysins-containing ultracentrifuged plasma from *B. glabrata*. The results include maps of the number of adhesion events and adhesion force (columns 1 and 2, respectively) and typical force-distance curves (column 3) measured on the retraction (red curves) of the tip from the cell surface. For the sake of completeness, force curves obtained on the approach of the tip (blue curves, column 3) are also reported.

**Figure 8:**
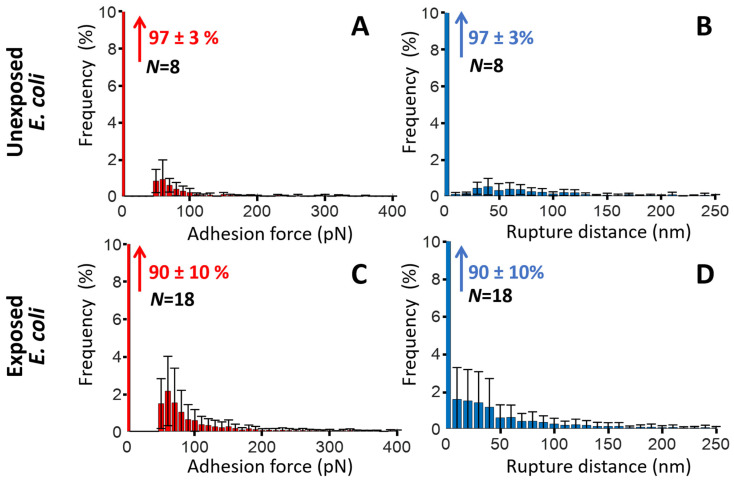
Distributions of the adhesion force and rupture distance for anti-biomphalysin (B1/2) antibody on *E. coli* before and after cell exposure to ultracentrifuged plasma from *B. glabrata*. Histograms showing the mean frequency distribution of the adhesion force (**A**,**C**) and (last) rupture distance (**B**,**D**) collected on *N* (specified) specimens of *E. coli* unexposed (**A**,**B**) and exposed (**C**,**D**) to biomphalysins-containing ultracentrifuged plasma from *B. glabrata.* The given numbers correspond to the percentage of force curves featuring the absence of adhesion between functionalized AFM tip and cell surface. The threshold force value for a proper detection of the adhesion peaks is 50 pN (**A**,**C**), and this choice is motivated by the signal-to-noise ratio in SMFS measurements (cf. details in Section 3.8).

For unexposed cells (Figure 7A), the force profiles reveal the absence of significant cell-to-tip adhesion, with a *quasi*-superposition of most of the approach and retraction force curves collected over the probed cell surface area. While this result confirms the expected absence of biomphalysin toxins at the surface of unexposed cells, the few adhesion events still identified in the SMFS curves probably result from *non-specific* electrostatic and/or hydrophobic interactions taking place between the anti-biomphalysin (B1/2) antibody functionalized tip and some compounds of *E. coli* cell wall like (lipo)polysaccharides. This result is supported by the histograms displayed in Figure 8A,B showing that ca. 97% of the force curves collected on *N* = 8 unexposed cells do not feature any adhesion to the functionalized tip, and the remaining 3% present adhesion force and rupture distance in the range ca. 50–100 pN and ca. 10–130 nm, respectively.

SMFS results obtained on *E. coli* cells after exposure to ultracentrifuged plasma (Figure 7B–D and Figure 8C,D) show a priori a contrasting picture. Indeed, while some of the selected exposed cells feature very few adhesion events (Figure 7D)—a result that is comparable to that for unexposed cells—others display multiple adhesion events with retraction force profiles marking either single or successive attachments/detachments of the tip to/from the cell surface (Figure 7B,C). The maps further show that the adhesion events—when present—appear rather randomly distributed over the scanned cell surface area. Obviously, like for unexposed cells, results demonstrate a certain heterogeneity in the response of exposed *E. coli* cells to the tip upon retraction (adhesion force ca. 50–200 pN, rupture distance ca. 10–150 nm, Figure 8C,D), and this heterogeneity operates at the scale of individual cells (non-homogeneous repartition of adhesion proxies within a given map) and from one cell to another (heterogeneity among maps, Figure 7B,D). The significance of the above adhesion properties of exposed *E. coli* must however be strongly nuanced. Indeed, the distribution histograms in Figure 8C,D show that only ca. 10% of the force curves measured on *N* = 18 exposed cells feature at least one event of adhesion to the anti-biomphalysin (B1/2) antibody functionalized tip.

A first order comparison between Figure 8A,C further highlights that cell exposure to biomphalysins-containing plasma has generated a ca. 7% increase in the amount of force curves displaying one or several adhesion events. As immunoblotting analysis demonstrated the absence of biomphalysins B1/2 on *E. coli* surface exposed to plasma (Figure 2), we can conclude here that the few tip-to-cell adhesion events identified for exposed *E. coli* bacteria originate from non-specific interactions, similarly to unexposed cells. The 7% increase in the amount of force curves displaying such interactions with the tip after cell exposure possibly results from a slight modification of, e.g., the hydrophobic/hydrophilic balance of *E. coli* surface (which includes possible modification of cell surface charge) following the adsorption of some *B. glabrata* plasma components. This explanation aligns with the fact that some plasma proteins can indeed bind to *E. coli* surface [32]. In turn, such an adsorption could very well increase the probability of non-specific interaction between cell and tip, in qualitative agreement with the distributions shown in Figure 8A,C. This scenario is further not incompatible with the absence of significant change in *E. coli* surface roughness after exposure to plasma (Figure 4E–H and Figure 5). The large error bars specified in the mean distributions given in Figure 8C,D prevents however from drawing more firm conclusions, and they illustrate the strong heterogeneities in cell response invoked above at the intra-individual and inter-individual scales.

**Figure 9:**
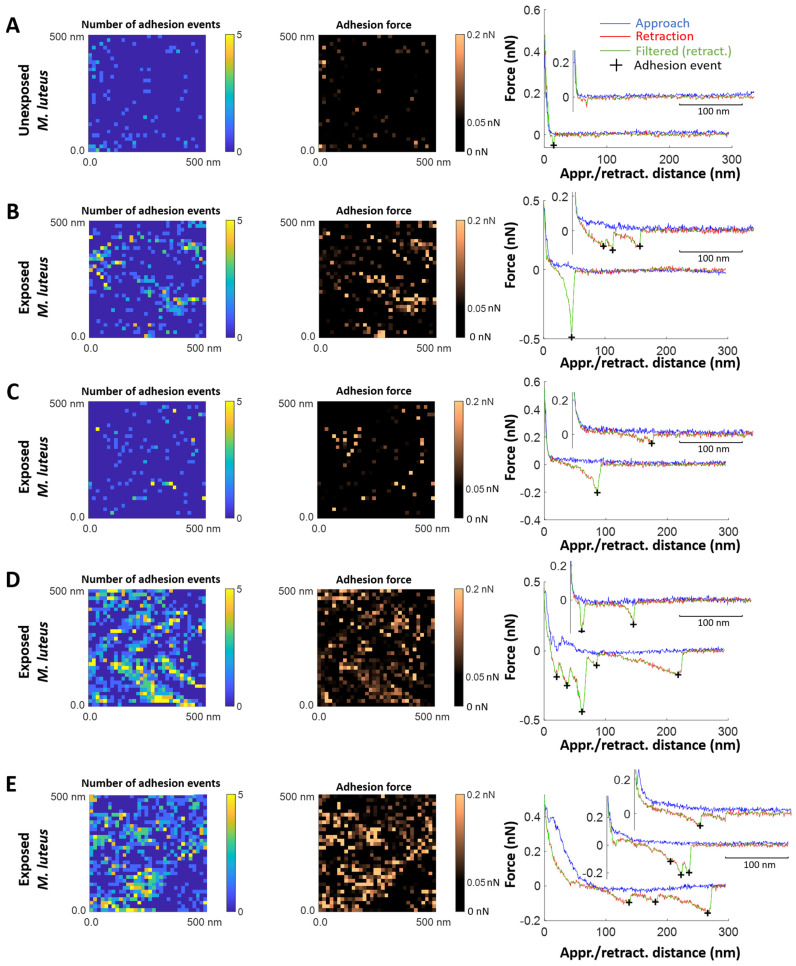
Adhesion properties of anti-biomphalysin (B1/2) antibody on *M. luteus* before and after exposure to ultracentrifuged plasma from *B. glabrata*. Representative SMFS measurements obtained in Force Volume mode with anti-biomphalysin (B1/2) antibody-functionalized AFM tip for different *M. luteus* cells unexposed (**A**) and exposed (**B**–**E**) to biomphalysins-containing ultracentrifuged plasma from *B. glabrata.* Meaning of columns 1 to 3 as in Figure 7.

**Figure 10:**
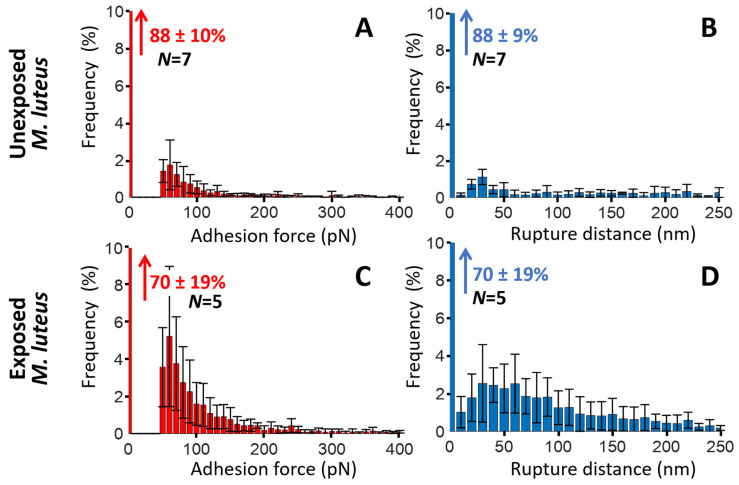
Distributions of the adhesion force and rupture distance for anti-biomphalysin (B1/2) antibody on *M. luteus* before and after cell exposure to ultracentrifuged plasma from *B. glabrata*. Histograms showing the mean frequency distribution of the adhesion force (**A**,**C**) and (last) rupture distance (**B**,**D**) collected on *N* (specified) specimens of *M. luteus* unexposed (**A**,**B**) and exposed (**C**,**D**) to biomphalysins-containing ultracentrifuged plasma from *B. glabrata.* The given numbers correspond to the percentage of force curves featuring the absence of adhesion between functionalized AFM tip and cell surface. The threshold force value for a proper detection of the adhesion peaks is 50 pN (**A**,**C**), and this choice is motivated by the signal-to-noise ratio in SMFS measurements (cf. details in Section 3.8).

Figure 9 and Figure 10 pertaining to *M. luteus* are the counterparts of Figure 7 and Figure 8 previously discussed for *E. coli.* As for *E. coli*, unexposed *M. luteus* also displays few adhesion events with anti-biomphalysin (B1/2) antibody functionalized tip (Figure 9A) and, for this bacterium type, it is now ca. 12% of the force curves collected on *N* = 7 specimens that feature a significant attractive interaction (ca. 50–150 pN) with the tip (Figure 10A, to be compared with the 3% value for ‘responsive’ unexposed *E. coli* cells). This difference can be probably explained by the distinct cell surface compositions of the Gram-negative *E. coli* and Gram-positive *M. luteus* bacteria and by the resulting change in the defining features of the operational non-specific attractive interactions: this is qualitatively supported by the differentiated distributions of rupture distances given for unexposed *E. coli* and *M. luteus* (Figure 8B and Figure 10B, respectively). The lipid content of Gram-negative bacterial envelope is significantly larger than that of Gram-positive cells (ca. 20% *vs.* 1–4%, respectively) and the surface charge of the former bacterial type is (in magnitude) generally lower than that of the latter [80]. Accordingly, the respective SMFS results on unexposed *E. coli* and *M. luteus* (Figure 8A,B and Figure 10A,B, respectively) suggest that their non-specific attractive interactions with the anti-biomphalysin (B1/2) antibody functionalized tip upon retraction are dominated by short-range electrostatic forces between zones of cell surface components and regions of anti-biomphalysin antibody via local coulombic and/or dipole–dipole interactions. Additional evidence for the absence of *specific* interactions between unexposed cells and tip is provided by the distribution of the rupture distances for unexposed *M. luteus* in the 0–250 nm range (Figure 10B). Indeed, this distribution is rather uniform with no sign of well-defined maxima that would be typically expected for systems where specific cell-to-tip interactions dominate [45,46,50,76,77].

Like for unexposed *E. coli*, the mean distributions of the adhesion force and rupture distances show a strong heterogeneity in cell-to-tip interaction data from the single cell to multi-individual levels.

The adhesion properties of exposed *M. luteus* cells significantly differ from those discussed so far for unexposed/exposed *E. coli* and unexposed *M. luteus*. Figure 9B–E and Figure 10C,D evidence indeed that ca. 30% of the force curves measured on *N* = 5 exposed *M. luteus* feature adhesion with the functionalized tip (to be compared with the 12% value for unexposed *M. luteus*, Figure 10A,B). The magnitude of the adhesion force now spans over a larger range of values (ca. 50–300 pN) as compared to unexposed *M. luteus*, a finding that is confirmed by the selected maps of adhesion force and the force profiles we report in Figure 9B–E for illustration purposes (columns 2 and 3, respectively). Overall, SMFS data unveil a larger density of cell surface pixels that feature 5 successive adhesion events (cf. yellow points in Figure 9D,E, column 1) and, contrary to previous examined situations, those adhesion events, when present in high number, seem to distribute according to patches over the probed cell surface. Most remarkably, despite of inherent heterogeneities of cell response already identified/discussed for *E. coli* and unexposed *M. luteus*, the frequency distribution of rupture distances for exposed *M. luteus* (Figure 10D) now takes the form of a well-defined monomodal bell shape, with a maximum around 30–60 nm. This is the signature of contributing specific cell-to-tip interactions, more precisely interactions between biomphalysins (B1/2) located at the surface of exposed *M. luteus* and anti-biomphalysin (B1/2) antibody grafted on the AFM tip. This result is consistent with the presence of biomphalysins B1/2 revealed on exposed *M. luteus* by immunoblotting assay (Figure 2) and by the changes in surface morphology and cell surface roughness due to interactions between *M. luteus* and biomphalysins (Figure 3, Figure 4 and Figure 5). Interestingly, specific protein–protein interactions measured by SMFS in the literature are typically in the range 20 to 150 pN or more [81,82,83]: this range fits well with the peak adhesion force of 60 pN revealed by Figure 10C and with the shift in the tail of the adhesion force distribution to values larger than 100 pN when switching from unexposed to exposed *M. luteus* case (Figure 10A and Figure 10C, respectively). The transition between these two cases is associated with a ca. 18% increase in the number of force curves that display adhesion events, most of them being the result of specific biomphalysin/anti-biomphalysin antibody interactions (cf. arguments above) and others probably not. It means that under the examined 5 min plasma exposure condition, a maximum of 18% cell surface coverage is achieved by biomphalysins. This low coverage is however apparently sufficient to generate significant cell surface damages and an increase in cell surface roughness (Figure 4 and Figure 5). Such an efficient action of biomphalysins is consistent with a patch-like repartition revealed by SMFS over the surface of exposed *M. luteus* because this repartition obviously minimizes efficiently the dilution of cell surface effects and maximizes biomphalysin action. This finding may further suggest a possible cooperativity in the action of biomphalysins, in line with the multiple adhesion events detected by the SMFS tip as a result of several specific interactions it would concomitantly experience when withdrawn from a given cell surface location (cf., e.g., Figure 9B,D). Other example of cooperative action of toxins includes that of β-Pore Forming Toxins inserted into membranes to form oligomeric transmembrane pores, which leads to cell permeation and possible lysis [84].

In summary, this is the first study that provides a multiscale assessment of the interactions operating between biomphalysin—a protein involved in the immune defense of *B. glabrata* against *S. mansoni* parasite—and Gram-negative *E. coli* and Gram-positive *M. luteus* bacteria, with the original recourse to Single Molecule Force Spectroscopy (SMFS) for probing interactions at the molecular and single cell levels. Cell aggregation assays, immunoblotting analysis, AFM imaging (and surface roughness evaluation) and SMFS measurements all consistently indicate the absence of specific interaction between biomphalysins B1/2 and *E. coli* (Table 1). On the contrary, exposure of *M. luteus* to biomphalysins-containing ultracentrifuged plasma affects cell surface integrity and leads to cell aggregation. The aggregation potential of biomphalysins B1/2 against *M. luteus* is positively correlated with the presence of a patch-like distribution of biomphalysin toxins specifically detected by SMFS over the surface of *M. luteus* with use of dedicated anti-biomphalysins (B1/2) antibody-functionalized AFM tip. The analysis of cell-to-tip SMFS force profiles and associated adhesion properties reveal that only a fraction of cell surface coverage (at most 18%) is needed to induce noticeable cell surface damage, which indicates a possible cooperativity in the action mode of biomphalysins.

The patchy distribution of BM on *M. luteus* suggests that its mechanism of action is localized rather than uniformly disrupting the entire cell surface. This could involve targeting specific components or regions of the cell wall. Future antimicrobial strategies could aim to enhance this localized damage, perhaps by designing molecules that preferentially bind to- or accumulate in- these BM-sensitive patches. The existence of BM-resistant patches on the cell surface hints at potential resistance mechanisms. These patches might lack the specific binding sites for BM or possess protective structures. New antimicrobials could be developed to target these resistant areas or to overcome the protective mechanisms present within them. Combining BM or BM-mimicking molecules with other antimicrobial agents that target different areas or mechanisms could be more effective. One agent could weaken the cell in BM-sensitive areas, making it more susceptible to another agent targeting the resistant patches. Furthermore, exploring if the BM-targeted domains on the *M. luteus* surface correspond to surface biomolecules that organize in patches, like teichoic acids (TA), could explain BM’s inability to bind Gram-negative bacteria which do not have TA on their surface.

As detailed in this work, biomphalysins B1/2 can induce damage to the *M. luteus* cell wall. Lysenin, another PFT belonging to the same aerolysin subfamily, produced by the earthworm *Eisenia fetida*, permeabilizes the cell membrane of another Gram-positive bacterium, *B. megaterium*. This permeabilization occurs without pore formation and exhibits a slower lysis rate compared to its effect on eukaryotic cells [31]. Moreover, lysenin gene transcription and synthesis increase in *E. fetida* cells challenged with the Gram-positive bacterium *Staphylococcus aureus*, but not with the Gram-negative *E. coli* [85]. Interestingly, Aep1, an aerolysin-like protein identified in zebrafish, is also active against Gram-positive bacteria such as *S. aureus*. The aep1 gene is constitutively expressed in immune-related tissues of adult zebrafish, and its expression is up-regulated upon bacterial infection [30]. While biomphalysin B1 was shown to interact with eukaryotic cells [18,19,32], the current results demonstrate that biomphalysins B1/2 can also directly interact with bacterial cell walls and possibly form oligomers in such an interaction context. This work further highlights the binding specificity of biomphalysins B1/2 towards two bacterial species, revealing a correlation between the binding and oligomerization of native plasmatic biomphalysins B1/2 and the subsequent alteration of their targeted cell surface. This correlation strengthens the argument that biomphalysins B1/2 are the primary lytic humoral effectors in the plasma of *B. glabrata*. All in all, these findings further support the role of aerolysin-like proteins in innate immune defense mechanisms against Gram-positive bacteria.

In frogs, the βγ-CAT complex —another host-derived aerolysin-like protein— protects against bacterial infection by forming pores in lysosomal membranes after endocytosis, triggering inflammasome activation and IL-1β release [86]. However, extrapolating this mechanism to biomphalysin remains highly speculative as no evidence currently supports similar intracellular trafficking or inflammasome involvement in *Biomphalaria glabrata*. As B1 was shown to bind and kill sporocysts of *S. mansoni* [18], the demonstration of its ability to bind the surface of a different cell type is of interest as it may imply a multi-target binding ability. This potential ability in binding different cell types coupled to its abundance in the plasma compartment may argue in favor of an immune sentinel role, providing plasma with a cell-lysis property against different intruders. It is noteworthy that biomphalysins are the only proteins found in *B. glabrata* plasma currently known to possess a lytic activity.

Overall, the results from this study provide insights into biomphalysin-bacteria interactions at various relevant scales from the molecule to single cell level, and they introduce some of the potentialities of single molecule force spectroscopy (SMFS) to decipher the molecular dialog between pathogens and immune factors of targeted cells. This work notably highlights the advantages of SMFS for investigating the presence and spatial distribution of biomphalysin toxin across the surface of bacteria at the molecular and single-cell levels. Furthermore, it illustrates how atomic force microscopy (AFM) offers insight into the potential damage inflicted by this toxin on the cell envelope, depending on exposure conditions and cell surface architecture (Gram-positive *versus* Gram-negative). Finally, the integration of AFM and multiparametric SMFS enables the assessment of the heterogeneity of cellular responses to biomphalysin at both intraparticle and multicellular levels. For the specific systems under study, limitations of SMFS technique include the contribution of non-specific interactions, which complicate the identification of specific cell interaction component with the anti-biomphalysin antibody-functionalized tip. Additionally, higher-resolved and potentially faster AFM-based techniques could be considered for imaging the potential formation of (pre)pores resulting from the interaction between the biomphalysin toxin and the cell surface. This would provide deeper insights into the mechanism of action of this toxin on bacterial targets.

Extensions of this work include the analysis of biomphalysin interactions with a broader range of pathogens, beyond *E. coli* and *M. luteus*, which could provide a more comprehensive understanding of BM target specificity. In addition, investigating by SMFS the effects of biomphalysin mutants featuring subtle variations in structure and/or composition could further help in dissecting the structure-function relationship of the toxin and identify critical steps involved in its interaction with bacterial surfaces. Moreover, the interpretation of experimental SMFS force curve measurements with computational molecular dynamics (MD) simulations could offer a powerful synergistic approach to tackle comprehensively biomphalysin action mode [87]. MD simulations could indeed provide atomistic-level details of the interactions between biomphalysin and bacterial surface components, thereby complementing the force measurements obtained by SMFS. Upon fitting theoretical models derived from MD simulations to the experimental force curves, one could gain a deeper understanding of the underlying energy landscapes, binding kinetics, and conformational changes involved in these interactions.

The current work on biomphalysin toxin falls within the framework of other broader applications of SMFS in microbiology, e.g., for unraveling pathogen resistance mechanisms, elucidating immune evasion strategies employed by microorganisms, or even informing the rational design and development of novel biomimetic antimicrobial agents. Concerning antibiotic resistance, a powerful approach consists of measuring directly the binding forces between an antibiotic and its target on a bacterial cell surface using an AFM tip functionalized with the antibiotic molecule. The observation of reduced binding forces in resistant bacterial strains compared to their susceptible counterparts can directly unveil the underlying molecular mechanisms of resistance. This strategy can involve mutations within the target protein, thus leading to a decreased affinity for the drug. This direct SMFS-based method could complement traditional biochemical assays by providing crucial spatial information on cell reactivity toward drugs and revealing potential heterogeneity in resistance mechanisms within a given bacterial population. In the realm of immune evasion, SMFS can be further employed to investigate the interactions between bacterial surface molecules and components of the host immune system, such as antibodies [54]. For instance, by attaching an antibody to the AFM tip and measuring its interaction force with a bacterial antigen, researchers can quantify the strength of the antibody–antigen interaction. Variations in these forces across different bacterial strains or under different environmental conditions can shed light on how pathogens evade immune recognition. Furthermore, SMFS can be used to study the mechanical disruption of bacterial membranes by antimicrobial peptides, providing insights into their mechanism of action and informing the design of more effective antimicrobial agents [71,72]. By pursuing these extensions and incorporating advanced computational techniques, the application of SMFS is expected to pave the way for innovative solutions to combat, e.g., infectious diseases and parasitosis.

## 3. Materials and Methods

### 3.1. Preparation of Biomphalysin-Containing Plasma

To obtain biomphalysin-containing plasma, hemolymph was collected and pooled from *B. glabrata* snails of the albino *BgBRE2* strain (Recife, Brazil), and centrifuged at 2000× *g* for 10 min at 4 °C so as to eliminate hemocytes. The plasmatic supernatant was then ultracentrifuged at 50,000 rpm for 2 h at 4 °C to eliminate hemoglobin (Optima L-90K Ultracentrifuge from Beckman Coulter using a SW 55 Ti rotor, Beckman Coulter, Brea, CA, USA).

### 3.2. Bacterial Cell Culture

Precultures of *M. luteus* (Gram-positive) and *E. coli* TOP10 and BL21DE3 (Gram-negative) were performed in 10 mL of lysogeny broth (LB) at 37 °C with shaking at 200 rpm overnight. The following day, the bacteria were cultured by adding 1 mL of preculture to 99 mL of LB medium, corresponding to a 1:100 dilution. Growth was stopped when the optical density (OD) reached 0.3 to 0.4 value at 600 nm. Cells were then harvested by centrifugation at 4500× *g* for 5 min to remove culture medium. This step was followed by two washes under the same conditions (4500× *g* for 5 min) using phosphate-buffered saline (PBS, Sigma, Darmstadt, Germany) for *M. luteus* and for *E. coli*. The pellets were resuspended in glucose and trehalose depleted Chernin’s Balanced Salt Solution (CBSS) [88], and OD was adjusted to 0.4 at 600 nm.

### 3.3. Cell Plasma-Exposure Conditions, SDS-PAGE and Western Blot Experiments

*M. luteus* and *E. coli* were cultured as detailed in the previous section. Each bacterial species was exposed either to ultracentrifuged plasma or kept in CBSS (unexposed condition) for 2 h at room temperature. Exposure was conducted with a bacterial suspension to plasma volume ratio of 1:1. This ratio was chosen to target conditions where the concentration of B1/2 is so significant that it is not a limiting factor. After three washes in phosphate-buffered saline solution (PBS, Sigma), exposed and unexposed bacteria were transferred in reducing Laemmli buffer and heat at 99 °C for 10 min for denaturation and sodium dodecyl-sulfate polyacrylamide (SDS-PAGE) gel analysis. Migration was performed in a 7.5% precast gel (Bio-Rad, Hercules, CA, USA) and Western blotting was conducted after PVDF (polyvinylidene difluoride) membrane transfer. Anti-biomphalysin 1 antibody was used and revealed with use of anti-rabbit HRP-tagged antibody (Invitrogen, Carlsbad, CA, USA). Anti-biomphalysin 1 antibody was designed on rabbit using a peptide (INALDRNDVNWADDA) and purified from rabbit serum on a peptide-coupled affinity column. Due to the high peptide sequence homology between biomphalysin 1 and 2 [19], only one amino acid is found to discriminate biomphalysin 1 and 2 within the selected immunogenic sequence, which makes the used antibody specific to both biomphalysin 1 and biomphalysin 2 proteins. Loading control was performed through a Schiff staining of the blotted membrane following the protocol by Thornton et al. (Figure S2 in Supplementary Materials) [89].

### 3.4. Cell Aggregation Assay

Bacterial cell aggregation assay was performed on a 96 wells plate, incubating *M. luteus* or *E. coli* with ultracentrifuged plasma or CBSS for 2 h at room temperature. Images were then captured using a Nikon Eclipse TS100 with a DS-Fi3 camera (Nikon Corporation, Tokyo, Japan) to address qualitatively the capability of cells to form aggregates under conditions of interest. As in Section 3.3, exposure was conducted with a bacterial suspension to plasma volume ratio of 1:1.

### 3.5. Cell Immobilization for Atomic Force Microscopy (AFM) Imaging and Single Molecule Force Spectroscopy (SMFS) Measurements

*M. luteus* cells were immobilized on a 1.2 µm microporous membrane (Isopore™ membrane, ref. RTTPO4700, Merck, Darmstadt, Germany). To that end, suspension of *M. luteus* dispersed in PBS was filtered using a vacuum filtration device to which the microporous membrane was attached. Three successive washes with PBS in Petri dishes were performed to remove excess bacteria and to retain only those cells which were trapped in the membrane pores. The microporous membrane was then carefully cut into 1 cm × 1 cm sections and attached to AFM measurement chamber using double-sided adhesive tape. *E. coli* cells were deposited on borosilicate glass slides. To optimize *E. coli* immobilization, slides were first pre-incubated with a 0.2% solution of cationic polymer (polyethyleneimine or PEI, Sigma, Mw = 750,000 g/mol) for 20 min, and then rinsed with a 1 mM KNO_3_ solution. Subsequently, 1 mL of *E. coli* suspension was deposited on a PEI-decorated slide for 20 min. The glass side was then rinsed with a 1 mM KNO_3_ solution to remove unbound or loosely bond *E. coli* bacteria. The slide supporting the adhered *E. coli* cells was then mounted in the AFM liquid cell prior to measurements.

### 3.6. Cell Imaging and Surface Roughness Evaluation by AFM, and SMFS Experiments

All experiments were performed with a FastScan Dimension Icon and NanoScope V controller (Bruker, Billerica, MA, USA) operated in either Peak-Force Tapping Mode for topography measurements (AFM imaging and cell surface roughness evaluation) or Force Volume mode for force spectroscopy experiments (SMFS measurements). All measurements were conducted at ambient temperature in PBS for *M. luteus* and in 1 mM KNO_3_ for *E. coli*, with a final volume of 4 mL. NPG10 tips (Bruker) with 30 nm curvature radius and 0.24 N·m^−1^ nominal spring constant (cantilever C) were used for cell imaging and cell surface roughness evaluation, and NPG10 tips (Bruker) with 30 nm curvature radius and 0.06 N·m^−1^ nominal spring constant (cantilever D) were selected for Force-Volume (FV) measurements. These spring constants are provided by the manufacturer. Prior to each measurement, refined values of spring constants were estimated following a calibration that consisted in determining the deflection sensitivity (nm/V) of the AFM tip via a force measurement taken on the rigid part of the substrate (microporous membrane or glass side substrate) and the subsequent application of the thermal tune method as detailed elsewhere [70,90]. All measurements were performed with a scan rate of 1 µm·s^−1^ at the apex of individual cells (500 nm × 500 nm probed cell surface area) selected on the microporous membrane or PEI-coated glass side substrate (relevant for *M. luteus* and *E. coli*, respectively).

***AFM imaging and cell surface roughness determination.*** To address the possible effects of biomphalysin on the structure and roughness of *M. luteus* and *E. coli* cell surfaces, Peak Force Tapping measurements were performed with the 0.24 N·m^−1^-NPG10 tips on a set of at least 9 cells for each bacterial species (immobilized as detailed above), before and after 5 min-exposure to 50 μL of biomphalysin-containing plasma (unless otherwise specified as in Figure S1). Prior to AFM measurements on cells pre-exposed to plasma, cells were washed with PBS (*M. luteus*) or 1 mM KNO_3_ (*E. coli*) to remove toxins that only weakly interacted with cell surface. AFM imaging measurements were also performed in the course of time to observe possible kinetics in the change in cell surface morphology and roughness induced by the toxin (Figure S1). Values of Root Mean Square (RMS) roughness of cell surfaces were systematically derived on 500 nm × 500 nm probed cell surface areas by means of NanoScope Analysis software 3.00 following a second-order flattening of the AFM image.

We are confident that the morphological patterns reported here for *M. luteus* and *E. coli* unexposed and exposed to biomphalysin (Figure 3 and Figure 4) correspond to true cell surface features for several reasons. First, as already reported in the literature [37,91], AFM imaging operated in liquid appears as one of the techniques of choice for high-resolution biological studies. Secondly, all precautions have been taken to ensure the best preservation of cell surface integrity upon imaging, i.e., (a) a careful preparation of the samples excluding any drying step, (b) the choice of an imaging mode based on intermittent cell-to-tip contact with a gently and precisely applied force, thereby limiting the potential damage of cells surface by the tip, and (c) well-controlled and limited post-treatment of the raw images with awareness of potential pitfalls. In this work, we have imaged the bacterial surface features in Peak-Force Tapping Mode with AFM tips and AFM operational parameters that ensure the best preservation of cell surface integrity upon scanning, as already reported in the literature and previous reports from our group [70,92,93]. In addition, the resolution achieved with the technique is of the order of the size of the AFM tip apex, i.e., 20–30 nm in radius.

***SMFS measurements.*** SMFS in Force Volume (FV) experiments consisted in monitoring, under liquid conditions and at room temperature, the interactions between an NPG10 tip (spring constant of 0.06 N·m^−1^) functionalized with an anti-biomphalysin 1/2 antibody (cf. details in Section 3.7) and the surface of individual *M. luteus* or *E. coli* cells. Prior to force measurements, immobilized *M. luteus* and *E. coli* bacteria were exposed for 5 min to 50 μL of biomphalysin-containing plasma collected along the lines defined in Section 3.1. After the 5 min exposure, the microporous membrane and glass sides supporting the immobilized *M. luteus* and *E. coli* cells, respectively, were abundantly washed with PBS and 1 mM KNO_3_, respectively, so as to remove the toxins that did not interact with the cells. During SMFS measurements, the functionalized AFM tip was approached towards- and subsequently retracted from- the surface of a selected immobilized cell at a constant speed of 1 µm·s^−1^ following a vertical ramp of 300 nm amplitude, and the setpoint adopted for all force measurements was 500 pN. The probed surface area of the cells was located at the cell apex in order to minimize AFM tip-to-sample surface convolution effects. Force measurements performed on single *M. luteus* and *E. coli* cell generated a map of 32 × 32 pixels over a 500 nm × 500 nm area of the analyzed cell surface, with each pixel corresponding to a pair of force *versus* distance curves measured upon approach and retraction of the functionalized tip to- and from- the cell surface, respectively (termed as *approach* and *retraction* force *vs.* distance curves, respectively, Figure 6). SMFS experiments were further performed in PBS and 1 mM KNO_3_ on *M. luteus* and *E. coli* cells, respectively, that were not exposed to biomphalysin-containing plasma (control experiments). Additionally, measurements with *non*-functionalized (bare) AFM tips were performed to ensure that the tip did not interact specifically with the cell surface after exposure to biomphalysin toxin.

### 3.7. AFM Tip Functionalization with Anti-Biomphalysin 1/2 Antibody

As mentioned above, SMFS measurements in FV mode were performed on *E. coli* and *M. luteus* using 0.06 N·m^−1^—NPG10 AFM tips functionalized with an anti-biomphalysin 1 (and 2) antibody, identical to that adopted for the Western blotting experiments. Preparation of the AFM tips started with their incubation in a UV-ozone chamber for 5 min to remove surface contaminants, followed by a wash with ethanol to ensure optimal cleaning. Next, two thiol solutions were prepared: one containing 16-mercaptohexadecanoic acid (solution A, Sigma-Aldrich, Darmstadt, Germany) and the other containing 11-mercapto1-undecanol (solution B, Sigma-Aldrich), both at a final concentration of 1 mM in absolute ethanol with a final volume of 10 mL each. A mixed solution of A and B was obtained with 0.5 mL of solution A and 4.5 mL of solution B, and the mixture was then homogenized by sonication for 1 min. AFM tips were then incubated overnight in this solution mixture to allow the formation of a self-assembled monolayer of thiols (SAM) over the surface of the AFM tips. After incubation, the tips were rinsed with ethanol and then dried with N_2_. The functionalization of the tips further proceeded with a chemical activation of the tip surface in NHS-EDS solution (40 mg of N-hydroxysuccinimide, NHS, and 100 mg of N-(3-dimethylaminopropyl)-N′-ethylcarbodiimide, EDS) dissolved in 4 mL of Milli-Q water for 30 min, followed by rinsing with Milli-Q water to remove the excess of reagents [94,95]. Finally, the activated AFM tips were incubated for 40 min with the anti-biomphalysin antibody, adjusted to a final concentration of 0.2 mg/mL in sodium chloride physiological solution. This ensured a covalent attachment of the antibody to the surface of the probe.

It is stressed that the grafting of biomolecules to an AFM tip does not significantly affect the physical properties of the probe (the so-called tip sensitivity and spring constant). To ensure that these properties were correctly configured, the functionalized tips were calibrated prior to each experiment, as specified in Section 3.6. As for the specificity, decorating the tip with anti-BM antibody is a mandatory step to specifically detect BM through antigen–antibody interactions. Here, we have chosen to covalently graft the anti-BM through the amines group of the proteins by NHS-EDS chemistry, thus ensuring a robust anchoring of the antibody to the tip. We cannot exclude that this grafting strategy somewhat modifies the free flexibility of the antibody, which would then slightly hinder BM detection. Despite of this uncertainty, the adopted grafting strategy does not affect the specificity of the recognition.

### 3.8. Processing of SMFS Interaction Measurements

The retraction force *vs*. distance curves provided by the NanoScope Analysis 3.00 software were processed by a homemade Matlab (R2022a) code calling for the ‘NSMatalbUtilities.m’ functions supplied by Bruker. The code was developed to identify—in every retraction force *vs.* distance curve measured at a given location (or pixel) of a selected individual cell surface—the range of vertical tip-to-surface distances marking the occurrence of adhesion event between the functionalized AFM tip and the cell surface material positioned at the pixel probed by the tip. The tip-to-sample adhesion corresponds to negative values taken by the force over the identified range of distances, as illustrated in Figure 6. The code then allowed an estimation of (i) the maximum amplitude of the force measured over the range of tip-to-sample distances where adhesion occurs, hereafter called ***adhesion force***, and (ii) the probe-to-sample distance at which the force abruptly decreases to zero value, hereafter called the ***rupture distance*** and defined by the separation distance at which the tip is detached from the cell surface (Figure 6). We empirically established a minimum threshold value of 50 pN for a proper detection of the adhesion peaks, and that value is motivated by the ca. 10 pN AFM measurement noise (slightly variable from one force curve to another) and the 3-sigma of that noise level [93]. The choice of the 50 pN threshold may exclude some of the adhesive peaks from the overall percentage of adhesion events detected per tested condition. However, this value was chosen based on a 3-sigma threshold to ensure that all the peaks correspond to true adhesive events rather that noise fluctuation, thereby minimizing the risk of false positives. Given the shapes of the gaussian distribution of the adhesion force events, for which all maxima are located above the 50 pN force-level, we are confident that the majority of the adhesion events are correctly considered in this work. Using a 3-sigma criterion is a common and accepted method in signal processing and data analysis to define a confidence interval that captures the majority of random noise (typically >99%) [93]. Prior to execution of the aforementioned processing steps, our developed Matlab code identified the baseline of the retraction force *vs.* distance curves, defined as the end time interval where the measured force varied linearly with the vertical position *z* of the AFM tip. This baseline then served as zero-force reference in force data postprocessing. When probing soft materials like cell surfaces in liquid, the dependence of the force on *z* in the last stage of the measurement can exhibit deviations from linearity. Consequently, an adaptive strategy for baseline identification was implemented. In this strategy, we assume that the maximum setpoint value of the force specified by the user is reached when the AFM tip has indented the cell envelope and the linear force *vs.* distance compliance regime is operational (Figure 6). Additional details on the procedure adopted to achieve (i) and (ii) are provided in Figure S3 in Supplementary Materials. As shown in Figure 6, the retraction force *vs.* distance curves featuring adhesion of the AFM tip to the cell surface can take the form of a sawtooth pattern [76,77,78,79]. The latter may be the result of a sequential unfolding of cell surface compounds like proteins that specifically bind the functionalized AFM tip and/or it may correspond to successive attachments and detachments of different cell surface components to and from the AFM tip when retracting from the cell surface (Figure 6). When relevant, the Matlab code could evaluate the **number of adhesion events** detected in each measured retraction-force *vs.* distance curve, that number being the number of local force maxima detected over the range of distances where there is adhesion (Figure 6). Also, the force corresponding to the very **last adhesion** event (or last peak adhesion force) prior to rupture was evaluated. In this work, the magnitude of the force associated with the **last adhesion** event systematically identified with the **adhesion force** defined above. The code was used to perform the above analysis of all 32 × 32 retraction force *vs.* distance curves measured over the 500 nm × 500 nm surface area of a given selected cell. In turn, spatial maps of the parameters relative to the tip-to-cell surface adhesion (e.g., adhesion force and number of adhesion events) were generated together with their frequency distributions presented in the form of histograms normalized by the total number of pixels probed on the selected cell surface area. In addition, mean statistical distributions of estimated adhesion parameters could be constructed by pooling data of different maps measured under a given condition (i.e., cells exposed or not to biomphalysin-containing plasma) on different *M. luteus* or *E. coli* originating from either the same or different cell cultures (Figure 8 and Figure 10). Here, again, the distributions were normalized by the total amount of pixels probed over the pooled spatial maps. For statistical analysis purposes, SMFS measurements with anti-biomphalysin antibody-functionalized AFM tip were carried on 5 to 18 *M. luteus* and *E. coli* cells, depending on the conditions of exposure to the biomphalysin-containing plasma.

## Figures and Tables

**Table 1:** Summary of some defining properties of *E. coli* and *M. luteus* surface prior to- and after- exposure to biomphalysin, as evaluated by AFM imaging and SMFS measurements. Details of bacteria-BM adhesion features are illustrated in Figure 7, Figure 8, Figure 9 and Figure 10 for *E. coli* and *M. luteus*, respectively.

	*E. coli*		*M. luteus*	
	Before Exposure	After Exposure	Before Exposure	After Exposure
Surface Roughness (nm)	3.0 ± 0.9 nm	2.9 ± 1.8 nm	2.1 ± 0.8 nm	4.3 ± 2.4 nm
% of SMFS force curves (or cell pixels) free of BM adhesion events	97 ± 3%	90 ± 10%	88 ± 10%	70 ± 19%

## Data Availability

The original contributions presented in this study are included in the article/supplementary material. Further inquiries can be directed to the corresponding author(s).

## Supplementary Materials

The following supporting information can be downloaded at https://www.mdpi.com/article/10.3390/toxins17060269/s1: Figure S1: Evolution of *M. luteus* surface morphology with time under constant condition of exposure to biomphalysin toxin. Figure S2: Schiff staining of immunoblotting membrane adopted in this work. Figure S3: Details on the constraints adopted for the detection of local maxima in the SMFS retraction force *vs.* distance curves.
