# Supplementary material for: Probing Bacterial Interactions with the Schistosoma mansoni-Killing Toxin Biomphalysin via Atomic Force Microscopy and Single Molecule Force Spectroscopy
Source: Toxins (Basel). 2025 May 27;17(6):269. doi: 10.3390/toxins17060269 (PMC12197742; doi:10.3390/toxins17060269)
Supplement: Supplementary file 1 [file toxins-17-00269-s001.zip › toxins-3619940-supplementary.pdf]

Supplementary Materials: Probing Bacterial Interactions with the *Schistosoma mansoni*-Killing Toxin Biomphalysin via Atomic Force Microscopy and Single Molecule Force Spectroscopy

Jihen Zouaoui, Pierre Poteaux, Audrey Beaussart, Nicolas Lesniewska, David Duval and Jérôme F.L. Duval

This document contains 3 Supplementary Figures (**Figures S1-S3**) and 3 pages. References mentioned in this document are listed on page 3.

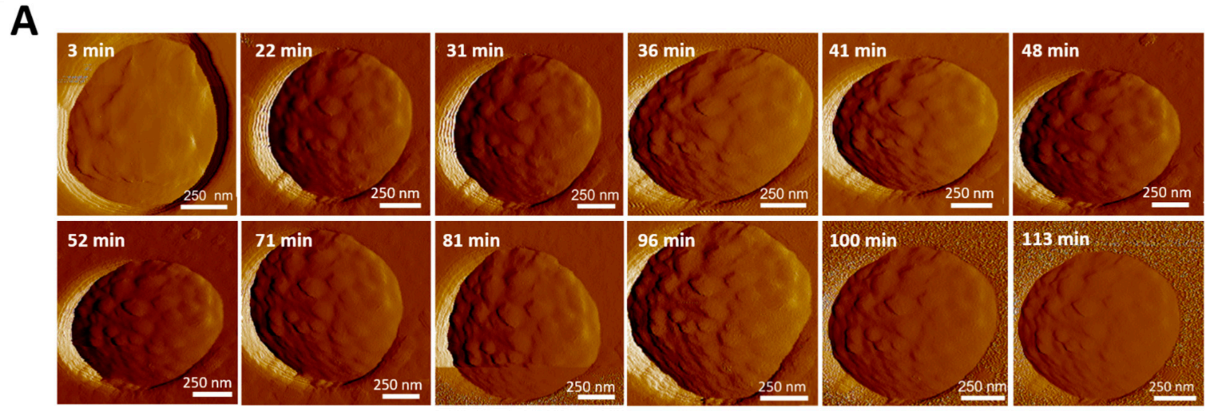

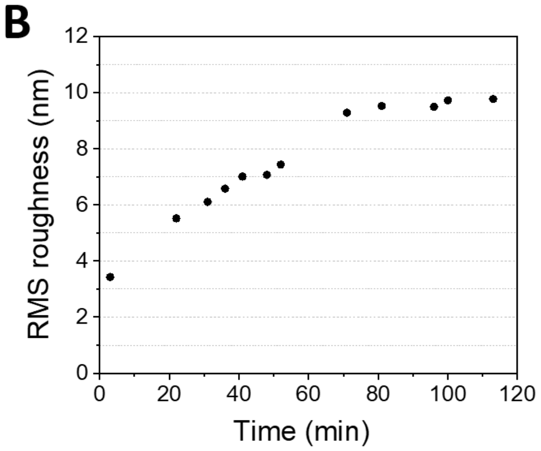

Figure S1. Evolution of *M. luteus* surface morphology with time under constant condition of exposure to biomphalysin toxin (4 mL PBS solution + 10 µL of biomphalysin-containing ultracentrifuged plasma). **(A)** Peak force error images of *M. luteus* bacteria (trapped in a porous membrane) at different times of exposure to the toxin (specified in white). The mixture 4 mL PBS solution + 10 µL of biomphalysin-containing ultracentrifuged plasma is injected at t=0 in the AFM set-up. **(B)** Graph showing the evolution of the RMS roughness of a *M. luteus* cell surface with time (RMS measured on 500 nm x 500 nm areas of the images displayed in panel A).

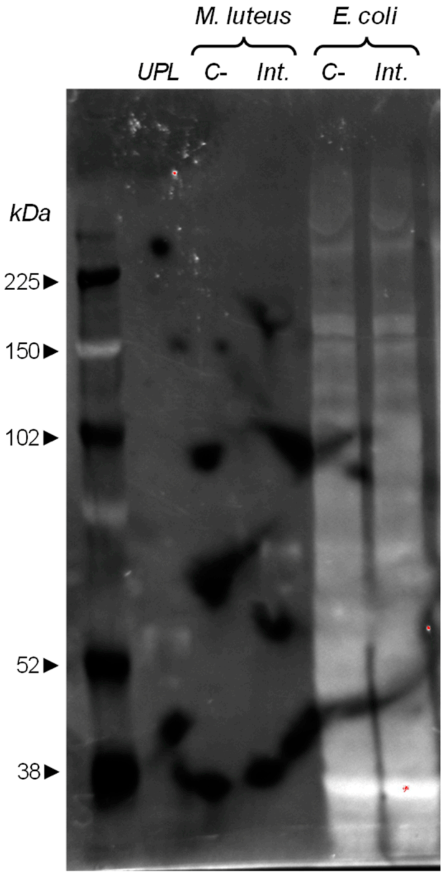

Figure S2. Schiff staining of immunoblotting membrane (cf. **Figure 2**) following the procedure by Thornton et al. [1]. Meaning of '*UPL*', '*C-*' and '*Int.*' is similar to that specified in **Figure 2** of the main text.

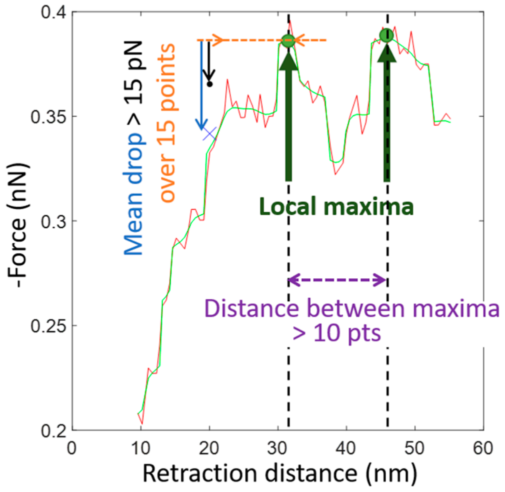

Figure S3. Zoom of a region of a representative retraction force curve (red curve) displaying two successive adhesion events (indicated by the green dots), and specification of the constrains adopted for their automatic detection based on the analysis of the (green) curve obtained by mean-shift smoothing of raw force data. The constrains pertain to the lowest acceptable values for (i) the distance (number of points) between successive local maxima upon retraction of the AFM tip from the cell surface, and (ii) the mean force-drop and number of points where that drop occurs for a given local adhesion event, as schemed in the figure.

Reference

1. Thornton, D.J.; Sheehan, J.K.; Carlstedt, I. Identification of glycoproteins on nitrocellulose membranes and gels. In *Basic Protein and Peptide Protocols*, 1st ed.; J.M. Walker, Ed.; in Methods in Molecular Biology™ series; Publisher Humana Totowa, NJ, Humana Press 1994; Volume 32, pp. 119-128 (doi: 10.1385/0-89603-268-X:119).
